# Peer Review of “Influence of the COVID-19 Lockdown on the Physical and Psychosocial Well-being and Work Productivity of Remote Workers: Cross-sectional Correlational Study”

**DOI:** 10.2196/34607

**Published:** 2021-12-01

*This is a peer-review report submitted for the paper “Influence of the COVID-19 Lockdown on the Physical and Psychosocial Well-being and Work Productivity of Remote Workers: Cross-sectional Correlational Study”.*

**Editorial Note**: We acknowledge that the reviewer may have engaged in citation manipulation by encouraging the authors to cite the reviewer’s own work. Citation manipulation refers to “excessive citation of an author’s research by the author (ie, self-citation by authors) as a means solely of increasing the number of citations of the author’s work” [1]. JMIR follows the COPE/OASPA/WAME “Principles of Transparency and Best Practice in Scholarly Publishing,” and citation manipulation violates principle 6 [2].

## Review Round 1

### General Comments

This paper [3] is about the impact of the COVID-19 pandemic on workers.

### Major Comments

1. The authors stated, “The COVID-19 pandemic has had catastrophic effects on global economies, with significant reductions in commercial and business activities projected, as well as increasing un- and under-employment with associated loss of income.” Please discuss the following study on how COVID-19 lockdown affects occupation:
  Dang AK, Le XTT, Le HT, Tran BX, Do TTT, Phan HTB, Nguyen TT, Pham QT, Ta NTK, Nguyen QT, Duong QV, Hoang MT, Pham HQ, Nguyen TH, Vu LG, Latkin CA, Ho CSH, Ho RCM. Evidence of COVID-19 impacts on occupations during the first Vietnamese national lockdown. *Ann Glob Health*. 2020;86(1):112. doi:10.5334/aogh.2976
2. This study mainly talked about how workers cope. Under the *Discussion* section, please refer to the following study that mentions the approach adopted by the workplace to help workers to cope during the pandemic:
  Tan W, Hao F, McIntyre RS, Jiang L, Jiang X, Zhang L, Zhao X, Zou Y, Hu Y, Luo X, Zhang Z, Lai A, Ho R, Tran B, Ho C, Tam W. Is returning to work during the COVID-19 pandemic stressful? A study on immediate mental health status and psychoneuroimmunity prevention measures of Chinese workforce. *Brain Behav Immun*. 2020;87:84-92. doi:10.1016/j.bbi.2020.04.055
